# Comparison between the Chemical Composition of Essential Oil from Commercial Products and Biocultivated *Lavandula angustifolia* Mill.

**DOI:** 10.1155/2023/1997157

**Published:** 2023-01-13

**Authors:** Velislava Todorova, Kalin Ivanov, Yoana Georgieva, Diana Karcheva-Bahchevanska, Stanislava Ivanova

**Affiliations:** Department of Pharmacognosy and Pharmaceutical Chemistry, Faculty of Pharmacy, Medical University-Plovdiv, 4002 Plovdiv, Bulgaria

## Abstract

The main aim of this study was to assess the differences in the chemical composition of essential oil from biocultivated *Lavandula angustifolia* in the Thracian Lowland floristic region, Bulgaria, and commercially available products from Bulgarian markets. Following the analytical results conducted with gas chromatography-mass spectrometry, we have established some differences in the chemical composition of the tested samples. The essential oil of biocultivated lavender contained 35 compounds, which represent 94.13% of the total oil. Samples from commercial products contained 28–42 compounds that represent 93.03–98.69% of the total oil. All the examined samples were rich in monoterpene hydrocarbons (1.68–12.77%), oxygenated monoterpenes (70.42–87.96%), sesquiterpene hydrocarbons (4.03–13.78%), and oxygenated sesquiterpenes (0.14–0.76%). The dominant components in all examined samples were linalool (20.0–45.0%) and linalyl acetate (20.79–39.91%). All the examined commercial samples contained linalool and linalyl acetate as was described in the European Pharmacopoeia, but in one of the samples, the quality of linalyl acetate is lower than that recommended in the European Pharmacopoeia.

## 1. Introduction

Lavender (*Lavandula angustifolia Mill.*) is one of the most popular aromatic plants in the Lamiaceae family with origin in the Mediterranean region and is cultivated worldwide for medicinal and commercial purposes [1, 2]. *Lavandula angustifolia* (*L. angustifolia*) has great economic values as an essential oil-producing plant. Because of its characteristic and pleasant aroma, as well as its therapeutic properties, the essential oil of lavender is of considerable importance in pharmaceutical, cosmetics, perfume, food, and flavor industries [3–5]. The floral essential oil of lavender is documented to have therapeutic effects such as antibacterial, antioxidant, antifungal, carminative, sedative, antidepressive, analgesic, and anti-inflammatory [3, 6, 7]. In addition, according to Basch et al., the aroma of lavender is one of the most widely utilized in aromatherapy, considered to be relaxing, with anxiolytic effects [8]. The multiple therapeutic applications of *L. angustifolia* are attributed mainly to the presence of volatile bioactive substances contained in the essential oil [4].

Lavender essential oil is usually produced by steam or hydrodistillation from flowering tops, and other used methods for oil extraction are supercritical CO_2_ fluid extraction, microwave, ultrasound, and turbohydrodistillation [9]. The chemical composition of the EO differs according to the extraction technique [5, 10]. However, essential oil from *L. angustifolia* is composed of various constituents, including esters: linalyl acetate, lavandulyl acetate, and geranyl acetate; alcohols: linalool, *α*-terpineol, and terpinen-4-ol; sesquiterpenes: *β*-caryophyllene; and monoterpene: cis-*β*-ocimene [2, 4, 10–13]. The greater proportion is linalyl acetate and linalool which are considered active constituents, but linalool is considered one of the most examined odorant molecules [14]. Both components are responsible for therapeutic effects. Moreover, all constituents contribute to the synergism of the total therapeutic effect. Today, lavender is cultivated around the world and enjoys continuing popularity for various therapeutic and cosmetic purposes [3, 12].

According to Stanev et al., Bulgaria has a long tradition of lavender cultivation and essential oil production dating since the 1900s [15]. Moreover, according to Stanev et al., Bulgarian lavender populations are characterized by high adaptability to the geographic, climate, and soil conditions of the country and consequently high essential oil content and quality [16]. This placed Bulgaria as one of the main lavender-growing and essential oil-producing countries, along with France [1, 13, 17]. Due to the economic value of lavender essential oil, interest in the development of new commercial products has increased. This study compares the chemical composition of essential oils from biocultivated *L. angustifolia* and commercial products in Bulgaria.

## 2. Materials and Methods

### 2.1. Chemicals and Reagents

For determination of retention indices (RIs), heptane (99%), octane (≥99%), nonane (99%), decane (≥99%), undecane (≥99%), dodecane (99%), tridecane (≥99%), tetradecane (≥99%), hexadecane (≥99%), heptadecane (99%), octadecane (99%), nonadecane (99%), and eicosane (99%) purchased from Merck KGaA (Darmstadt, Germany) were used. For diluting essential oils, hexane purchased from Thermo Fisher Scientific GmbH (Bremen, Germany) was used.

### 2.2. Plant Material and Oil Extraction

*L. angustifolia* was cultivated in the area of Belashtitsa, Thracian Lowland floristic region, Bulgaria. The cultivated plant has grown in a continental climate with an average annual temperature of 12.3°C and rendzina soil. The essential oil was obtained from the air-dried flowering tops of lavender by hydrodistillation using the Clevenger apparatus for 3 h according to the standard procedure described in the European Pharmacopoeia 9 (07/2018:1534) [18]. After completion of distillation, the collected oil was dried over anhydrous sodium sulfate and stored in dark glass vials at 4°C until GC-MS analysis.

### 2.3. Chromatographic Conditions

Gas chromatography-mass spectrometry (GC-MS) was used for the analysis. GC-MS analyses were carried out using Bruker Scion 436-GC SQ MS, Bremen, Germany. The column used was a Bruker BR-5 ms fused silica capillary (0.25 *μ*m film thickness and 15 m × 0.25 mm i.d.). The oven temperature was initially held at 45°C for 1 min and then increased to 140 at 3°C/min, and after that, it was increased to 250°C at 17°C/min and then held for 1 min. The flow rate of helium (carrier gas) was 1 mL/min. The injector split ratio was 1 : 50, and the injection volume was 1 *μ*L. The range of *m*/*z* was 50–350 in the full-scan mode. To compare the spectral data and retention indices of compounds, the Wiley NIST11 Mass Spectral Library (NIST11/2011/EPA/NIH) and the literature were used. Retention index values were calculated and compared with reported values for a C7–C20 series of *n*-alkane standards.

## 3. Results and Discussion

**Figure 1:**
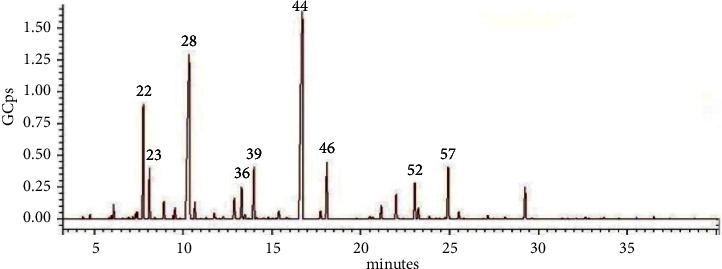
Chromatogram of the cultivated *L. angustifolia* essential oil. The numbers are related to 22, trans-*β*- ocimene; 23, cis-*β*-ocimene; 28, *β*-linalool; 36, (-)-terpinen-4-ol; 39, *α*-terpineol; 44, linalyl acetate; 46, lavandulyl acetate; 52, caryophyllene; 57, cis-*β*- farnesene.

The distilled lavender EO had a strong odour of linalool and linalyl acetate. Its colour was yellow. The extracted essential oil and commercial essential oils were diluted with hexane and analyzed with GC-MS.  Figure 1 shows the chromatogram of the EO of the cultivated lavender.

The essential oil of the cultivated lavender contained 35 compounds, which represent 94.13% of the total oil. Samples from commercial products contained 28–42, which represent 93.03–98.69% of the total oil.  Table 1 shows the chemical composition found in essential oils from biocultivated *L. angustifolia* (CLA) and those from commercial products (CP 1–7).

The essential oil obtained from the biocultivated lavender was characterized by monoterpene hydrocarbons (11.33%), oxygenated monoterpenes (72.94%), and sesquiterpenes hydrocarbons (8.47%). Among the monoterpene hydrocarbons, the main components were trans-*β*- ocimene (6.70%) and cis-*β*-ocimene (2.89%). Oxygenated monoterpenes were detected in higher amounts. The prevailing components from oxygenated monoterpenes were linalyl acetate (31.46%), *β*-linalool (23.13%), lavandulyl acetate (4.21%), *α*-terpineol (3.95%), and (-)-terpinen-4-ol (2.28%). The presence of linalool and linalyl acetate in lavender essential oil determined its anti-inflammatory, cytotoxic, antimicrobial, repellent effect, sedative, local anaesthetic, analgesic, antioxidant, antimicrobial, and other activities [14, 19–22]. The main volatile components of sesquiterpene hydrocarbons were caryophyllene (2.87%) and cis-*β*-farnesene (3.99%).

All analyzed commercial essential oils were rich in monoterpene hydrocarbons (1.68–12.77%), oxygenated monoterpenes (70.42–87.96%), sesquiterpenes hydrocarbons (4.03–13.78%), and oxygenated sesquiterpenes (0.14–0.76%). The representatives of the monoterpenes with the highest content were trans-*β*- ocimene (0.29–7.19%) with higher amounts in CP 3 and cis-*β*-ocimene (0.15–4.06%) with higher amounts in CP 7. Oxygenated monoterpenes, especially *β*-linalool (24.34–35.99%) and linalyl acetate (20.79–39.91%), were in higher amounts in all of the commercial products. They were followed by (-)- terpinen-4-ol (1.54–5.34%), lavandulyl acetate (0.41–3.87%), and eucalyptol (0.79–5.38%). The sesquiterpene hydrocarbons caryophyllene (1.96–6.09%), cis-*β*-farnesene (0.08–3.78%) and trans-*β*-farnesene (0.14–6.85%) were also isolated. Oxygenated sesquiterpenes (0.14–0.76%) were in the lower concentration in all of the analyzed commercial products.

According to the European Pharmacopoeia, the relative content of lavender oil compounds should be in the following ranges: limonene (maximum 1.0%), 1,8-cineole (maximum 2.5%), 3-octanone (0.1–5.0%), camphor (maximum 1.2%), linalool (20.0–45.0%), linalyl acetate (25.0–47.0%), terpinen-4-ol (0.1–8.0%), lavandulyl acetate (minimum 0.2%), lavandulol (minimum 0.1%), and *α*-terpineol (maximum 2.0%) [18]. Only one sample contained more than 1% lemonene, and the rest corresponded to the requirements of the European Pharmacopoeia. In our results, only in two of the commercial products, the amount of 1,8-cineole was higher than 2.5%. According to the requirements of the European Pharmacopoeia, isolated 3-octanone was in the described ranges in all of the examined samples, but in two of the examined samples (CP 2 and CP 6), camphor did not correspond to the requirements. All the samples contained the recommended amounts of linalool and terpinen-4-ol. Only sample 2 contained less than 25% linalyl acetate. Not all the samples corresponded to the requirements for lavandulyl acetate, lavandulol, and *α*-terpineol.

The obtained results can be compared with these by other researchers. In research conducted with Ukrainian cultivars, Pokajewicz et. al. found that linalool (11.42–44.05%), (-)-terpinen-4-ol (1.17–11.25%), and linalyl acetate (15.79–35.27%) as the main chemical compounds [23]. Moreover, Dong et al., researchers from China, reported linalool (19.71%), linalyl acetate (26.61%), *α*-terpineol (3.61%), and lavandulyl acetate (12.68%) as the main compounds [24]. In addition, Adaszynska et al., researchers from Poland, reported that linalool (15.85–23.88%) and linalyl anthranilate (1.58–12.78%) are the main ingredients together with geraniol acetate (2.37–10.61%), caryophyllene (2.78–6.24%), 1-terpinen-4-ol (5.53–9.73%), and p-menth-1-en-8-ol (3.98–7.94%) [25].

The difference in the composition of lavender essential oils may be due to influence of cultivation methods and different geographic regions on the accumulation of chemical compounds [26–28]. According to Bara, some of the exogenous factors such as light and soil (pH and constituents) may increase the concentration of terpenes [29]. It is considered that many enzymes of secondary pathways are UV-B-dependent [29, 30]. Hassiotis et al. reported that temperature and the flowering stage have a positive influence on the EO composition, but rainfall during the flowering period has a negative influence on EO content [31]. The linalool content is influenced by temperature, flower development, and rainfalls, and rainfalls during the harvest period decrease linalool production [31]. Also, the addition of synthetic compounds would affect the differences in the chemical composition and its concentrations [6, 32]. Moreover, according to Filly et. al., the different isolation methods of essential oil may also lead to differences in composition [33]. For further studies, it is recommended conducting a survey on adding synthetic and part synthetic compounds to commercial products containing essential oils. The method used for analyses of the essential oil chemical profile is also important, and the GC-MS analysis represents well separation and identification of volatile compounds [34]. The method described above could also be used for further analysis.

## 4. Conclusions

A total of 50 volatile compounds were found in lavender biocultivated essential oil, which represents 93.17% of the total oil. The following terpene classes were found in the essential oil from lavender biocultivated essential oil: monoterpene hydrocarbons (11.33%), oxygenated monoterpenes (72.94%), and sesquiterpene hydrocarbons (8.47%). Oxygenated monoterpenes were detected in higher amounts. The prevailing components from them were linalool (23.13%) and linalyl acetate (31.46%). Volatile compounds found in the commercial products were 28–42, which represent 93.55–98.69% of the total oil. Commercial products were rich in oxygenated monoterpenes, and especially, linalool (24.34–35.32%) and linalyl acetate (20.79–39.91%) were in higher amounts. The results of this study indicate that the essential oil content and quality of the analyzed commercial products corresponded to the recommendations given in the European Pharmacopoeia.

## Figures and Tables

**Table 1:** The GC data for essential oil components identified in cultivated *L. angustifolia* and commercial products, where tr indicates traces, less than 0.05%.

No.	Compound	RI	Class	CLA	CP 1	CP 2	CP 3	CP 4	CP 5	CP 6	CP 7
1	*α*-Pinene	937	MH	0.14	0.35	0.14	0.21	0.11	0.22	0.55	0.31
2	Camphene	947	MH	0.2	tr	0.17	0.17	tr	0.19	0.39	0.19
3	*β*-Pinene	967	MH	tr	—	—	tr	0.11	0.11	0.38	tr
4	1-Octen-3-ol	973	O	0.10	tr	0.33	0.16	0.21	0.26	0.15	0.20
5	3-Octanone	976	O	0.16	0.13	1.75	0.29	0.20	0.44	tr	1.38
6	*β*-Myrcene	980	MH	0.74	0.11	0.48	0.43	0.55	0.34	0.42	0.65
7	3-Octanol	988	O	—	—	0.66	—	—	—	tr	0.43
8	3-Carene	993	MH	—	—	tr	0.13	—	tr	—	0.13
9	Acetic acid, hexyl ester	1001	O	0.1	1.86	0.73	0.34	0.13	0.26	0.16	0.69
10	*p*-Cymene	1003	MH	tr	0.54	—	tr	—	0.25	0.14	tr
11	o-Cymene	1007	MH	0.13	—	0.24	0.18	tr	0.10	—	0.20
12	Limonene	1010	MH	0.33	—	1.21	0.55	0.33	0.40	0.74	0.93
13	Eucalyptol	1013	MO	0.42	5.38	1.58	0.86	2.17	0.79	3.57	0.90
**14**	***Trans*-*β*- ocimene**	**1022**	**MH**	**6.70**	**0.29**	**1.21**	**7.19**	**4.93**	**3.87**	**1.46**	**5.81**
**15**	**cis-*β*-ocimene**	**1031**	**MH**	**2.89**	**0.15**	**1.21**	**2.47**	**1.86**	**3.84**	**0.37**	**4.06**
16	*γ*-Terpinene	1039	MH	tr	0.11	0.13	0.15	tr	0.11	0.14	0.30
17	Linalool oxide	1051	MO	1.10	—	0.21	0.16	tr	0.33	0.19	0.23
18	*α*-Terpinolene	1064	MH	0.20	0.14	tr	tr	0.11	tr	0.29	0.14
**19**	***β*-Linalool**	**1087**	**MO**	**23.13**	**34.04**	**35.99**	**24.34**	**35.32**	**26.60**	**24.68**	**26.92**
20	1-Octen-3-yl acetate	1095	O	1.01	—	0.46	0.68	0.36	0.76	0.25	0.82
21	Camphor	1123	MO	0.34	2.78	0.47	0.29	0.13	0.46	8.29	0.28
22	Isoborneol	1140	MO	—	0.64	—	—	—	—	—	—
23	Lavandulol	1150	MO	—	—	2.34	—	1.64	—	—	1.68
24	Endo-borneol	1151	MO	1.68	1.62	—	1.44	—	2.44	2.82	—
25	**(-)-Terpinen-4-ol**	**1161**	**MO**	**2.28**	**2.15**	**2.64**	**3.97**	**1.54**	**3.36**	**3.48**	**5.34**
26	Cryptone	1166	MO	0.37	—	0.35	0.28	—	—	—	0.22
**27**	***α*-Terpineol**	**1179**	**MO**	**3.95**	**0.66**	**1.29**	**0.98**	**—**	**0.75**	**0.64**	**1.15**
28	Cis-geraniol	1214	MO	0.17	—	0.16	tr	0.21	0.10	—	0.13
29	Cuminal	1215	MO	—	—	0.20	0.15	—	tr	—	—
**30**	**Linalyl acetate**	**1249**	**MO**	**31.46**	**39.91**	**20.79**	**36.97**	**35.39**	**36.03**	**36.47**	**30.79**
31	Bornyl acetate	1273	MO	0.57	0.26	—	0.22	0.13	0.14	tr	0.15
**32**	**Lavandulyl acetate**	**1282**	**MO**	**4.21**	**0.41**	**3.57**	**3.87**	**2.88**	**3.67**	**2.40**	**3.53**
33	Nerol acetate	1359	MO	1.01	tr	0.32	0.31	0.43	0.12	0.14	0.28
34	Geranyl acetate	1380	MO	1.85	0.11	0.51	0.51	0.81	0.44	0.23	0.45
35	Zingiberene	1440	SH	—	—	0.18	tr	tr	tr	0.11	tr
**36**	**Caryophyllene**	**1407**	**SH**	**2.87**	**4.64**	**5.15**	**5.72**	**3.85**	**6.09**	**1.96**	**4.06**
37	*α*- Santalene	1412	SH	0.87	tr	—	0.69	0.44	0.78	0.33	0.50
38	Trans-*α*-bergamotene	1427	SH	0.18	—	0.17	0.20	0.12	0.20	—	0.13
39	Humulene	1442	SH	Tr	0.28	0.13	0.14	tr	0.20	tr	tr
**40**	**Cis-*β*-farnesene**	**1454**	**SH**	**3.99**	**0.23**	**tr**	**3.26**	**2.77**	**—**	**—**	**3.78**
41	Trans-*β*-farnesene	1455	SH	—	—	6.85	0.14	—	2.39	1.37	—
42	Gemacrene D	1468	SH	—	—	0.89	0.61	0.59	—	tr	0.69
43	*β*-Copaene	1469	SH	0.56	tr	—	—	—	—	tr	—
44	*γ*-Cadinene	1490	SH	—	—	0.26	0.29	tr	0.25	0.26	0.19
45	*β*-Sesquiphellandrene	1510	SH	—	—	0.15	—	tr	0.14	—	tr
46	Caryophyllene oxide	1563	SO	—	0.15	0.50	0.34	0.14	0.65	0.10	0.26
47	Tau-cadinol	1607	SO	tr	—	0.13	tr	—	0.11	0.17	tr
48	*α*-Bisabolol	1615	SO	—	tr	—	—	—	—	0.38	—

*Terpene classes*											
	Monoterpene hydrocarbons (MHs)			11.33	1.68	4.79	1148	8.00	9.43	4.88	12.77
	Oxygenated monoterpenes (MOs)			72.94	87.96	70.42	74.35	80.45	75.23	82.91	72.05
	Sesquiterpene hydrocarbons (SHs)			8.47	5.15	13.78	11.05	7.77	10.05	4.03	9.35
	Oxygenated sesquiterpenes (SOs)			—	0.15	0.63	0.34	0.14	0.76	0.65	0.26
	Others (O)			1.37	1.99	3.93	1.47	0.90	1.72	0.56	3.52
	Total identified			94.13	96.93	93.55	98.69	97.26	97.19	93.03	97.95

The results show the mean values of the three independent samples of each sample, CLA and CP 1–7. The standard error of the mean does not exceed 2% of it and has been removed to simplify reporting.

## Data Availability

All data generated and analyzed during this study are included in the manuscript.
